# Neighborhood Characteristics and Mental Health From Childhood to Adolescence

**DOI:** 10.1001/jamanetworkopen.2025.4470

**Published:** 2025-04-10

**Authors:** Niloofar Shoari, Marta Blangiardo, Monica Pirani

**Affiliations:** 1 MRC Centre for Environment & Health, Department of Epidemiology and Biostatistics, Imperial College London, London, United Kingdom; 2 Great Ormond Street Institute of Child Health, Population, Policy and Practice Research and Teaching Department, University College London, London, United Kingdom

## Abstract

**Importance:**

The relationship of neighborhood environmental and socioeconomic factors with mental health across childhood and adolescence remains unclear.

**Objective:**

To investigate the associations of neighborhood characteristics with mental health at various developmental stages, from early childhood to late adolescence.

**Design, Setting, and Participants:**

This population-based cohort study used data from the Millennium Cohort Study, including approximately 19 000 children born in the UK from 2000 to 2002. This analysis included individuals in England with complete data on neighborhood exposures at ages 3, 5, 7, 11, 14, and 17 years. Data were analyzed from January to December 2023.

**Exposures:**

Neighborhood-level air pollution, green space, and socioeconomic status.

**Main Outcomes and Measures:**

Strengths and Difficulties Questionnaire (SDQ) scores linked with exposure to air pollution, green space, and socioeconomic status, measured at participants’ residential addresses, were analyzed using a hierarchical bayesian regression model. Hypotheses were formulated after data collection.

**Results:**

The sample included 3595 children and adolescents, with 1826 (50.5%) female; 3012 participants (83.8%) were White and 583 participants (16.2%) were another ethnicity. The mean (SD) SDQ score was 7.1 (5.1). After adjusting for individual and household factors, neighborhood socioeconomic status emerged as the strongest factor associated with mental health. Residing in affluent neighborhoods was associated with improved mental health, with this association magnifying through adolescence. For children of the same age, those in the most affluent neighborhoods had log-transformed SDQ scores 0.73 (95% credible interval [CrI], 0.58 to 0.88) lower at age 17 years compared with their peers in the most deprived areas; at age 5 years, scores were 0.31 (95% CrI, 0.17 to 0.45) lower. Exposure to particulate matter with diameter less than 2.5 μm was associated with poorer mental health during early childhood (age 3 years), with an effect size of 0.15 (95% CrI, 0.08 to 0.22). While green space was not directly associated with improved mental health, a sex-based difference was observed, with males showing more favorable associations (interaction, −0.10 [95% CrI, −0.17 to −0.03]).

**Conclusions and Relevance:**

In this cohort study of children and adolescents, associations between neighborhood characteristics and mental health evolved from childhood through adolescence. These findings suggest that targeted interventions in disadvantaged neighborhoods and strategies to protect young children from air pollution are essential. A comprehensive approach is recommended to incorporate air pollution, green space, and socioeconomic status not only in residential neighborhoods but also in other settings, such as schools.

## Key Points

**Question:**

Are neighborhood characteristics associated with children’s mental health at different developmental stages?

**Findings:**

In this cohort study of 3595 children and adolescents in England followed up for 17 years, neighborhood socioeconomic status was associated with mental health, especially during adolescence. Exposure to air pollution at age 3 years was associated with poorer mental health, whereas green space was not associated with mental health.

**Meaning:**

This cohort study enhances understanding of how neighborhood characteristic are associated with mental health, emphasizing the combination of environmental and socioeconomic factors and how these associations change with age.

## Teaser

This cohort study investigates the associations of neighborhood characteristics with mental health at various developmental stages, from early childhood to late adolescence.

## Introduction

Childhood and adolescent mental health issues, including anxiety, depression, eating disorder, and self-harm, are growing concerns in the UK, with probable mental disorder estimated to affect approximately 18% of young people as of 2023.^1^ Early mental health issues profoundly affect educational attainment,^2,3^ social relationships,^4^ and long-term health.^5^ While the social aspects of neighborhoods are well-studied in relation to mental health,^6,7,8^ less is known about the influence of environmental characteristics.

Air pollution and green space are key environmental factors linked to mental health. Childhood exposure to air pollution has been associated with depression,^9^ behavioral problems,^10^ attention-deficit/hyperactivity disorder,^11,12^ and later-life mental and neurological disorders.^13,14,15,16,17^ Similarly, air pollution exposure during adolescence is associated with an increased risk of mental disorders.^14,18,19^

Research on green space and mental health shows mixed results. Some studies report positive associations,^8,20,21,22,23,24^ while others indicate null or adverse effects.^25,26,27,28,29^ Inconsistencies may stem from differences in confounders, exposure characterization and assessment windows, and the timing of mental health outcomes. For example, research exploring air pollution and green space individually vs jointly report varying findings.^9,30,31,32,33^

Neighborhoods influence mental health through multiple pathways, including direct environmental exposures, shared socioeconomic conditions, and broader contextual effects.^34^ Our study examines contextual environmental exposures, namely air pollution and green space, while simultaneously capturing area-level socioeconomic characteristics that reflect shared vulnerabilities within neighborhoods. However, gaps remain regarding the timing of exposure and its effects across the lifespan. Research often averages exposures over time^31,35,36^ or examines specific age at exposure,^9,17,19,27,30,32,36,37,38,39,40,41^ overlooking potential developmental windows of heightened sensitivity. Identifying critical age windows when neighborhood exposures are associated with mental health is crucial for developing age-appropriate interventions and public health strategies.

This study uses data from the Millennium Cohort Study (MCS) to address these gaps, examining neighborhood-level environmental factors associated with mental health across 17 years. We investigate whether specific age windows show stronger associations, and we assess variations by sex, ethnicity, and socioeconomic status. Finally, we explore whether green space characteristics, such as greenness or functionality, are associated with mental health.

## Methods

The MCS was approved by the National Health Service research ethics committee system and ensured informed parental consent and child agreement for participation. This cohort study adhered to the Strengthening the Reporting of Observational Studies in Epidemiology (STROBE) reporting guideline for cohort studies.

### Design and Setting

We conducted a longitudinal analysis of children and adolescents in England from age 3 to 17 years. The study leveraged repeated measures of exposures and outcomes across multiple time points, allowing us to capture both within-individual changes over time and cross-sectional associations at specific ages.

### Population

The MCS is a nationally representative UK-based cohort of children born between September 2000 and January 2002. Its primary aim is to investigate the social, economic, and health factors associated with early life and development. Sampling was stratified by country and the type of electoral ward, with oversampling of socioeconomically disadvantaged and ethnically diverse areas, as described by Plewish et al.^42^ Data collection occurred over 8 sweeps, from infancy to young adulthood. Specifically, cohort members were surveyed at approximately ages 9 months (sweep 1), 3 years (sweep 2), 5 years (sweep 3), 7 years (sweep 4), 11 years (sweep 5), 14 years (sweep 6), 17 years (sweep 7), and 23 years (sweep 8). Data were collected through face-to-face interviews with parents, teachers, and self-completed questionnaires. Ethnicity was collected through self-report by the parent (usually mother) at the time of the child’s enrollment into the study. In this study, we categorized ethnicity as White and other ethnicity (eg, Black or Black British, Caribbean, Indian, Pakistani or Bangladeshi, or multiple ethnicities) to facilitate interpretation of findings. Cohort members participated in data collection at ages 14, 17, and 23 years. The original dataset included 18 293 children from across the UK. For this analysis, we focused exclusively on children who resided in England throughout their participation in the study.

For this analysis, we used data from sweeps 1 to 7 (infancy to age 17 years),^43,44,45,46,47,48,49^ reconstructing neighborhoods of residence using lower layer super output areas (LSOAs). The LSOAs are geographic units used in UK census data, each representing approximately 1500 residents. In cases of multiple births, we used data on first-born twins and triplets only.

The overall MCS population showed stable or expected variations in individual and household characteristics over time, such as socioeconomic status and family structure (eTable 1 in Supplement 1). Our analysis focused on a subset of 3595 cohort members who had complete residential histories across sweeps 1 to 7. Starting with 2868 children and adolescents with complete LSOA records, the sample was expanded to 3595 by inferring no relocation if the same LSOA was recorded before and after a sweep with missing data. Exposures were assigned to each member based on the LSOA recorded at each sweep. Participation varied across sweeps due to missing data or dropout. Inclusion and exclusion criteria are presented in eFigure 1 in Supplement 1.

### Mental Health

Mental health was assessed using the Strength and Difficulties Questionnaire (SDQ), a validated screening tool measuring emotional and behavioral difficulties.^50^ The SDQ demonstrates strong psychometric properties, with good internal consistency and test-retest reliability.^51^ The SDQ comprises 20 items grouped into 4 scales (emotions, conduct, hyperactivity/inattention, and peer relationships), scored from 0 to 40. Higher SDQ score indicates greater mental health challenges. SDQ scores were collected at ages 3, 5, 7, and 14 years with the parent-reported questionnaires, at age 11 years with a teacher-reported questionnaire, and at age 17 years with both parent- and self-reported questionnaires. To ensure result consistency, only parent-reported SDQ scores were considered, thus excluding data from age 11 years.

In this study, we analyzed the total difficulties score to capture overall mental health status. Subscales were not separately analyzed, as our focus was on general mental health rather than specific behavioral problems. SDQ scores were treated as a continuous measure, without applying clinical cutoff points.

### Covariates and Exposures

At each survey sweep, we measured exposure to green space, air pollution, and neighborhood socioeconomic status based on the LSOA of residence, analyzing both exposures and outcome for the same period. This approach allowed us to assess how these environmental and socioeconomic factors evolved over time and were associated with mental health trajectories.

#### Green Space

We used 2 metrics for green space: Normalized Difference Vegetation Index (NDVI; 2000-2017), derived from National Aeronautics and Space Administration Moderate Resolution Imaging Spectroradiometer and reflecting neighborhood greenness, and the area of green space (2017-2018) based on its designated function, including the public parks, gardens, and playing fields, sourced from Ordnance Survey.^52^ Hereafter, we refer to *green space* as both NDVI and the area of green space unless specified otherwise.

#### Air Pollution

Annual averages of nitrogen dioxide (no_2_), particle matter with less than 10 and less than 2.5 µm (PM_10_ and PM_2.5_, respectively) for 2000 to 2018 were obtained from the Department for Environment, Food and Rural Affairs.^53^ More details are reported in eMethods 1 in Supplement 1.

#### Socioeconomic Status

Neighborhood socioeconomic status was measured by the 2004 Index of Multiple Deprivation (IMD), which combines 7 weighted domains of deprivation: income, employment, education, health, crime, barriers to housing and services, and living environment. More details are in eMethods 2 in Supplement 1. The first decile of the IMD score indicates the most deprived areas and the tenth decile, the least deprived. In our study, we presented distributions of IMD by grouping them into 3 categories: 1 to 3 deciles, most deprived; 4 to 7 deciles, moderately deprived; and 8 to 10 deciles, least deprived. This categorization aimed to simplify analysis and interpretation.

Household income and neighborhood IMD data were not collected in sweep 7, leading to additional missing information. To address this, we assumed income stability from the previous sweep. Additionally, we included only cohort members who did not relocate between sweep 6 and sweep 7, allowing us to assign them the IMD deciles from sweep 6.

We included several individual-, household-, and neighborhood-level covariates based on existing literature and data availability. At the individual level, we considered sex, ethnicity (White or other ethnicity), obesity (normal weight, overweight, obese), and whether the cohort member reported a longstanding illness. Obesity classification was based on categories in the dataset, which were derived from body mass index. Household-level variables included poverty status (below or above 60% median equivalized income), maternal education level (National Vocational Qualification) equivalent, mother ever diagnosed with depression or serious anxiety, and family structure (single or couple-parent carer). Only maternal education at birth and diagnosis of maternal mental health issues at birth were available from sweep 1. At the neighborhood level, we included rural vs urban classification. For a more in-depth explanation of the covariates, see eMethods 3 in Supplement 1 and cohort documentation.^43,44,45,46,47,48,49^

### Statistical Analysis

We specified a hierarchical Bayesian regression model to accommodate the nested structure of the longitudinal data. We modeled log-transformed SDQ test score as a function of individual-, household-, and neighborhood-level characteristics. Individuals-specific random intercepts accounted for within-individual dependency across repeated SDQ scores, and time-varying covariates for neighborhood factors identified critical age of exposure from early life to adulthood. The variance inflation factor score was used to detect potential multicollinearity.

Let *Y_it_* denote the log-transformed SDQ test score for participant *i* (*i* = 1, 2, …, 3595) measured at age *t (t* = 3, 5, 7, 14, and 17, corresponding to sweep 2, 3, 4, 6, and 7, respectively). We assumed that *Y_it_* follows a normal distribution, as specified below:

*Y_it_ ~ Normal (μ_it_,σ^2^)*

*μ_it_ = α_i_ + ∑_p_β_p_x_it,p_ + ∑_k_γ_t,k_z_it,k_ + interaction*

*α_i_ ~ N(μ_α_,σ^2^_α_)*

*γ_t,k_ ~ N(μ_γ,k_,σ^2^_γ,k_).*

Here, *α_i_* is the individual-specific intercept, modeled by a normal distribution with mean *μ_α_* and variance σ*^2^*_α_.The term *∑_p_β_p_x_it,p_* captures the effects of individual- and household-level variables *x_it,p_*, each associated with a corresponding coefficient *β_p_*, where the set of coefficients is given by β_1_, β_2_, *…*, *… β*_p_. The term *∑_k_γ_t,k_z_it,k_* includes *k* time-varying neighborhood-level covariates *z_it,k_*, each linked to a coefficient γ = γ*_t,1_*, γ*_t,2_*, γ*_t,3_*, γ*_t,k_*. For the fixed-effects *β*, and hyperparameters *μ_α_* and *μ_γ_* we specified a *Normal*(0,1000) prior. For the variance parameters σ^2^, σ^2^_α_, and σ^2^_y,k_ we assumed an inverse-gamma (1,0.01) prior. This is equivalent to specifying a prior on precision (τ = 1/σ^2^) under the common bayesian framework, where *τ* represents the reciprocal of the variance. We explored effect modification with sociodemographic factors by including various interaction terms between sex, ethnicity, and poverty and neighborhood-level exposures. We considered a prior *Normal*(0,1000) for the coefficient of the interaction terms.

The bayesian model was implemented using the Markov Chain Monte Carlo methods via the R package NIMBLE version 1.3.0 (R Project for Statistical Computing),^54^ with 2 chains of 10 000 postburn iterations after a 10 000 iteration burn-in. Convergence was assessed via trace plots and the Gelman-Rubin statistic.

We iteratively modeled neighborhood-level factors, first assessing air pollution, green space, and socioeconomic status individually, then examining their combined effects, exploring shifts in coefficients by neighborhood socioeconomic status. Model selection was guided by the Watanabe-Akaike information criterion to balance model fit and complexity.^55^

Sensitivity analyses evaluated model robustness by (1) comparing models with missing vs complete data, (2) testing the of time-varying coefficients independence, (3) examining time-varying neighborhood effects independently, (4) modeling SDQ score with a Poisson distribution, (5) using alternative hyperpriors distributions, (6) restricting to urban areas only, and (7) limiting the sample to cohort members with complete LSOA records. Additionally, we ran a model that included only the cumulative exposure variables that is typically used to assess long-term health outcomes. We then compared it with the main model to examine whether cumulative exposure masks age-specific associations and explore potential differences in outcomes. Significance was determined as a 95% credible interval (CrI) that did not cross zero. Data were analyzed from January to December 2023.

## Results

Among 3595 children and adolescents included in this study, 1826 (50.5%) were female; 3012 participants (83.8%) were White and 583 participants (16.2%) identified as another ethnicity. The mean (SD) SDQ score was 7.1 (5.1). The Table shows selected characteristics of our final study sample, while eTable 1 in Supplement 1 compares these characteristics with the original population data for England. The maps of distribution of environmental characteristics, including air pollution, greenness, and the area of green space, across England are shown in eFigure 2 in Supplement 1.

**Table.** Descriptive Statistics of the Sample in England at Each Sweep

Characteristic	Patients by survey sweep, No. (%)					
	2 (n = 3353)	3 (n = 3566)	4 (n = 3570)	5 (n = 3516)	6 (n = 3459)	7 (n = 3392)
Total SDQ score, mean (SD)	8.7 (4.8)	6.3 (4.4)	6.5 (4.9)	4.6 (4.7)	7.2 (5.6)	6.7 (5.5)
SDQ score components, mean (SD)						
Emotional problems	1.8 (1.3)	1.2 (1.5)	1.4 (1.7)	1.2 (1.8)	1.7 (2.0)	1.7 (2.2)
Conduct problems	2.5 (1.9)	1.2 (1.3)	1.1 (1.4)	0.5 (1.1)	1.1 (1.5)	0.9 (1.4)
Hyperactivity/inattention	3.5 (2.3)	2.9 (2.2)	3.0 (2.4)	1.9 (2.3)	2.6 (2.3)	2.1 (2.2)
Peer problems	1.4 (1.5)	0.9 (1.3)	1.0 (1.5)	1.0 (1.6)	1.5 (1.8)	1.5 (1.8)
Sex						
Male	1641 (48.9)	1753 (49.2)	1757 (49.2)	1735 (49.3)	1734 (50.1)	1692 (49.9)
Female	1712 (51.1)	1813 (50.8)	1813 (50.8)	1781 (50.7)	1725 (49.9)	1700 (50.1)
Ethnicity^a^						
White	2810 (83.8)	2989 (83.8)	2993 (83.8)	2944 (83.7)	2899 (83.8)	2843 (83.8)
Other ethnicity	543 (16.2)	577 (16.2)	577 (16.2)	572 (16.3)	560 (16.2)	549 (16.2)
Chronic illness						
Yes	517 (15.4)	679 (19.0)	659 (18.5)	473 (13.5)	576 (16.7)	578 (17.0)
No	2836 (84.6)	2887 (81.0)	2911 (81.5)	3043 (86.5)	2883 (83.3)	2814 (83.0)
Obesity						
Normal	2619 (78.1)	2869 (80.5)	2908 (81.5)	2625 (74.7)	2611 (75.5)	2471 (72.8)
Overweight	574 (17.1)	553 (15.5)	496 (13.9)	703 (20.0)	630 (18.2)	619 (18.2)
Obese	160 (4.8)	144 (4.1)	166 (4.6)	188 (5.35)	218 (6.3)	302 (8.9)
Household characteristics						
OECD equivalized household income, mean (SD), £	413 (240.2)	428 (233.4)	461.7 (238.7)	492.2 (174.8)	490.1 (174.2)	NA
Family status						
2 Parents/carers	3064 (91.4)	3165 (88.8)	3115 (87.3)	2935 (83.5)	2845 (82.2)	2651 (78.2)
1 Parent/carer	289 (8.6)	401 (11.2)	455 (12.7)	581 (16.5)	614 (17.8)	741 (21.8)
Mother diagnosed with depression/anxiety at birth						
Yes	2666 (79.5)	2808 (79.0)	2813 (78.8)	2776 (79.0)	2729 (78.9)	2700 (79.4)
No	687 (20.5)	746 (21.0)	757 (21.2)	740 (21.0)	730 (21.1)	701 (20.6)
Poverty						
≥60% Median poverty	2763 (82.4)	2913 (81.7)	3004 (84.1)	3139 (89.3)	2981 (86.2)	2940 (86.7)
<60% Median poverty	590 (17.6)	653 (18.3)	566 (15.9)	377 (10.7)	478 (13.8)	452 (13.3)
Mother education at birth, NVQ level						
1	353 (10.5)	379 (10.6)	383 (10.7)	381 (10.8)	370 (10.7)	348 (10.3)
2	1272 (37.9)	1351 (37.9)	1348 (37.8)	1320 (37.5)	1301 (37.6)	1270 (37.4)
3	402 (12.0)	419 (11.7)	421 (11.8)	417 (11.9)	406 (11.7)	405 (11.9)
4	1134 (33.8)	1211 (34.0)	1212 (33.9)	1197 (34.0)	1177 (34.0)	1169 (34.5)
5	195 (5.8)	206 (5.8)	206 (5.8)	201 (5.7)	205 (5.9)	200 (5.9)
Neighborhood characteristics, mean (SD)						
PM_2.5_, μg/m^3^	14.1 (2.3)	12.0 (2.0)	11.1 (1.9)	11.1 (1.6)	9.3 (1.3)	9.4 (1.8)
PM_10_, μg/m^3^	21.4 (3.3)	18.1 (3.0)	16.7 (2.4)	15.1 (2.0)	13.9 (2.0)	14.4 (2.5)
no_2_, μg/m^3^	20.7 (8.0)	19.9 (7.5)	18.6 (7.2)	17.9 (6.5)	14.8 (6.2)	13.9 (5.7)
NDVI	0.5 (0.1)	0.5 (0.1)	0.5 (0.1)	0.5 (0.1)	0.6 (0.1)	0.5 (0.1)
Green space, mean (SD), ha	5.8 (30.1)	6.2 (30.8)	5.9 (22.2)	6.2 (20.1)	6.4 (22.8)	6.4 (22.8)
IMD decile						
1-3 (Most deprived)	923 (27.5)	934 (26.2)	893 (25.0)	816 (23.2)	787 (22.8)	757 (22.2)
8-10 (Least deprived)	1082 (32.3)	1213 (34.0)	1257 (35.2)	1289 (36.7)	1301 (37.6)	1289 (38.0)

Abbreviations: ha, hectares; IMD, Index of Multiple Deprivation; NDVI, Normalized Difference Vegetation Index; no_2_, nitrogen dioxide; NVQ, National Vocational Qualification; OECD, Organization for Economic Co-operation and Development; PM_2.5_, particulate matter with diameter less than 2.5 μm; PM_10_, particulate matter with diameter less than 10 μm.

^a^ Including Black or Black British, Caribbean, Indian, Pakistani or Bangladeshi, or multiple ethnicities.

The SDQ scores were right-skewed (eFigure 3 in Supplement 1), reflecting all scores collected over the study period, and showed a general decrease from early childhood to late adolescence, with a slight increase observed during early adolescence (Table). We observed a consistent decrease in mean exposure to neighborhood air pollution from 2004 (at age 3 years) to 2017. Specifically, the mean (SD) exposure levels at each sweep were 14.1 (2.3), 12.0 (2.0), 11.1 (1.9), 11.1 (1.6), 9.3 (1.3), and 9.4 (1.8) μg/m^3^ for PM_2.5_; 21.4 (3.3), 18.1 (3.0), 16.7 (2.4), 15.1 (2.0), 13.9 (2.0), and 14.4 (2.5) μg/m^3^ for PM_10_; and 20.7 (8.0), 19.9 (7.5), 18.6 (7.2), 17.9 (6.5), 14.8 (6.2), 13.9 (5.7) μg/m^3^ for no_2_. All these concentration levels remained above the World Health Organization’s recommended annual averages, which are 5 μg/m^3^ for PM_2.5_, 15 μg/m^3^ for PM_10_, and 10 μg/m^3^ for no_2_.^56^ During the same period, exposure to neighborhood green space showed a modest increasing trend.

We present results from the best-fit model, with sensitivity analyses provided in eResults 2 in Supplement 1. Figure, A shows the estimated coefficients β for individual and household covariates. Figure, B displays neighborhood-level exposures γ at ages 3, 5, 7, 14, and 17 years. Individual-level factors, such as male sex, overweight or obesity (compared with normal weight), and a long-standing illness were associated with higher log-transformed SDQ scores, suggesting poorer mental health. The difference in log-transformed SDQ scores between children of White ethnicity and those of other ethnicities was small, with the 95% CrI including zero.

**Figure.**
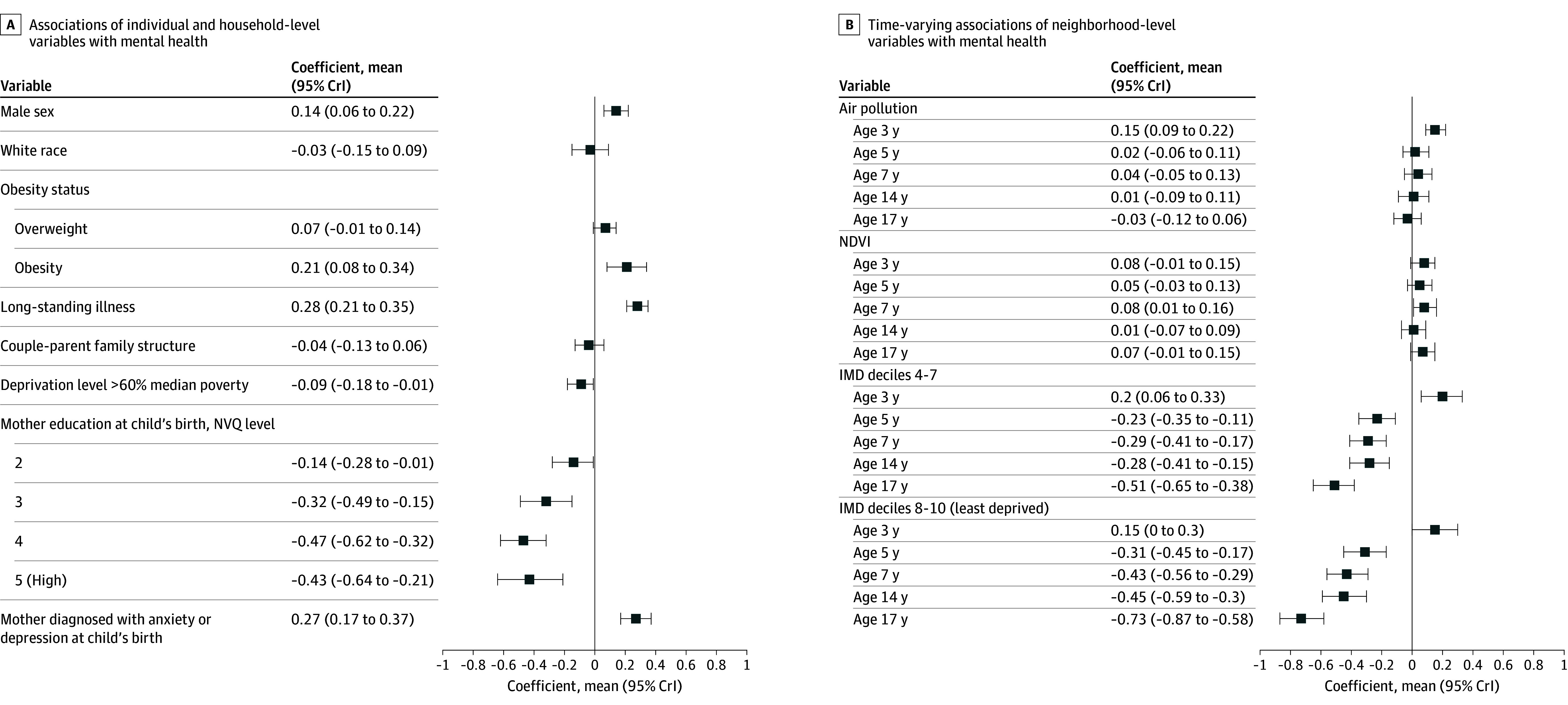
Estimated Regression Coefficients of the Main Model With Particulate Matter Less Than 2.5 μm (PM_2.5_) The results for Index of Multiple Deprivation (IMD) are compared with deciles 1 to 3. CrI, credible interval; NVQ, National Vocational Qualification.

At the household level (Figure, A), there was no difference between 2-parent and single-parent households. Children with families above the 60% median poverty threshold showed lower log-transformed SDQ scores, although with some uncertainty. Maternal educational was negatively associated with log-transformed SDQ scores, with higher education associated with better mental health for their offspring. In contrast, diagnosed maternal anxiety or depression at birth was associated with higher log-transformed SDQ scores, suggesting poorer child mental health.

In terms of age-dependent associations with neighborhood characteristics, exposure to all air pollutants was associated with higher log-transformed SDQ score, primarily at age 3 years (Figure, B). Specifically, each 1-μg/m^3^ increase of exposure at age 3 years was associated with log-transformed SDQ score increases of 0.15 (95% CrI, 0.09-0.22) for PM_2.5_, 0.16 (95% CrI, 0.09-0.22) for PM_10_, and 0.09 (95% CrI, 0.02-0.16) for no_2_. The associations of exposure to air pollution at ages 5, 7, 14, and 17 years with SDQ score were negligible and included zero. Neighborhood greenness (as measured with NDVI) showed an unexpected positive association with SDQ scores, although with high uncertainty, and the area of green space showed no discernable association with mental health.

Residing in more affluent neighborhoods (higher IMD deciles compared with the most deprived deciles 1-3) had a protective association that increased gradually with age. For example, at age 7 years, we found a difference of −0.43 (95% CrI, −0.56 to −0.29) in the log-transformed SDQ score compared with living in the most deprived neighborhood. However, this difference became notably larger at age 17 years, with a mean difference of −0.72 (95% CrI, −0.88 to −0.58) for those in the affluent neighborhoods. Surprisingly, the estimated coefficient for neighborhood IMD was unexpectedly positive at age 3 years, diverging from the overall trend.

When the model coefficients were compared with those of the cumulative exposure model for neighborhood factors, the coefficient estimate for air pollution was notably weakened and showed higher uncertainty (eResults 1 in Supplement 1). This suggests that cumulative exposures may mask potential age-specific associations.

The associations of neighborhood greenness with SDQ varied by sex, with males showing lower log-transformed SDQ score as neighborhood greenness increased (difference, −0.10 [95% CrI, −0.15 to −0.03]). However, this association diminished when the area of green space was considered instead of greenness. Other interactions did not substantially affect model fit. When the air pollutants were specifically PM_10_ and no_2_, the estimated coefficients were very similar to those shown in the Figure; these results are presented in eFigure 4 and eFigure 5 in Supplement 1. The trace plots showing the model convergence are displayed in eFigure 6 in Supplement 1.

### Interplay of Air Pollution, Green Space, and Deprivation

Examining green space (as measured with NDVI) alone revealed modest, uncertain negative associations across all age groups. In contrast, air pollution was the sole time-varying variable consistently associated with higher log-transformed SDQ scores across all age windows, with the strongest association observed at age 3 years.

Our analysis of the associations of neighborhood deprivation consistently found that children residing in moderately to least-deprived neighborhoods (IMD deciles 4-10) had lower log-transformed SDQ scores compared with those living in the most deprived neighborhoods (IMD deciles 1-3). This association intensified with age, emphasizing the evolving association of neighborhood socioeconomic status with mental health.

Including neighborhood socioeconomic status in the model accounted for the associations of environmental exposures, except for air pollution at age 3 years, where the association remained positive, although attenuated. Coefficients from single-exposure models are in eTable 2 in Supplement 1, along with sensitivity analysis models and results.

## Discussion

In this cohort study, we identified age windows when the associations of neighborhood characteristics and socioeconomic status with mental health differed, contributing to a better understanding of how neighborhood environments are associated with mental health during different developmental stages. While numerous studies have explored these associations among children and adolescents,^57,58^ fewer have investigated how neighborhood environmental exposures are associated with mental health.

### Neighborhood Socioeconomic Status and Mental Health

Our findings suggest that socioeconomic factors are more strongly associated with mental health outcomes than the environmental characteristics, aligning with previous studies.^59,60^ Adolescents in affluent neighborhoods had better mental health compared with those in deprived areas, likely due to better amenities, fewer stressors, and stronger social support.^61,62^ In contrast, adolescents in deprived neighborhoods are more likely to be exposed to stressors like violence and crime.^63,64^ Additionally, adolescence is a crucial period for identity formation, where socioeconomic disparities become more apparent.^65^

We observed an unexpected lower log-transformed SDQ score at age 3 years among cohort members living in neighborhoods with IMD deciles 4 to 10 with to the most deprived areas. This may be attributed to unobserved and unmeasured covariates (eg, parenting choices) that were not accounted for in this study.

### Air Pollution and Mental Health

The association between air pollution and mental health was strongest during early childhood, particularly at age 3 years. This aligns with clinical evidence showing that exposure to air pollution in early life, particularly from conception to age 5 years, leads to neurological changes, including alteration in brain white matter and volume due to neuroinflammation triggered by air pollutants.^66,67^ As children age, the association weakens, possibly due to lower vulnerability in adolescence or less time spent in residential neighborhoods.^7^

The reduced association between air pollution and mental health observed at older ages could be partially attributed to decreased air pollution levels over time. This highlights the significant role of air pollution in mental health outcomes and underscores the importance of continued efforts to reduce exposure, as even modest improvements in air quality may contribute to better mental health.

### Green Space and Mental Health

Contrary to expectations, we found no meaningful association between green space and mental health. This may reflect the modest associations of green space when analyzed alongside air pollution and socioeconomic status or limitations in our green space metrics, which did not capture quality, accessibility, or functionality.^68^ Other studies have similarly noticed that the presence of both air pollution and green space variables can modify or negate each other’s effect, highlighting the importance of examining their joint impact.^69,70^ For example, using the MCS data, Mueller and Fluori^71^ found no direct association between green space and children’s self-regulation when the model was adjusted for neighborhood air pollution as well as child- and family-level covariates.

We observed that the associations of neighborhood green space varied by sex, with males experiencing greater mental health benefit. This may suggest that males are more vulnerable to neuroinflammatory responses triggered by air pollution,^72^ making greenness exposure particularly beneficial for them. Our findings highlight the importance of considering sex differences green space-related health research.

### Combined Associations of Multiple Neighborhood Exposures With Mental Health

The associations between environmental exposures and mental health should be interpreted cautiously, as these exposures are intertwined with other neighborhood characteristics. Through iterative modeling adjustments, we found that neighborhood deprivation was the most consistently associated factor, overshadowing the associations of environmental exposures. However, the association between air pollution and mental health in children aged 3 years remained consistently positive despite the attenuation.

### Strengths and Limitations

This study has some strengths. Our nationwide sample enabled us to assess mental health and neighborhood characteristics with high spatial resolution, contributing to understanding time windows of vulnerabilities to neighborhood-level exposures. However, potential selection bias and unmeasured confounders, such as parental decisions, criminal records, parenting skills, school exposures, household smoking, activity patterns, green space quality, and crime rates, may affect our findings. Descriptive statistics revealed overrepresentation of children from White ethnic backgrounds, 2-parent families, and less-deprived neighborhoods. However, the hierarchical bayesian regression model with individual-specific random effects helps mitigate concerns about confounding factors.

This study also has some limitations. Misclassification of exposure due to our reliance on residential LSOA data and lack of activity pattern information (eg, commuting, leisure activities, or time spent indoors vs outdoors) may have influenced the observed associations. Additionally, assuming financial stability between sweep 6 (age 14 years) and 7 (age 17 years) may introduce bias by overrepresenting families with greater residential stability. Furthermore, including only the firstborn child in cases of multiple births may introduce bias, as birth order is associated with health and developmental outcomes.

## Conclusions

This cohort study found that socioeconomic characteristics of neighborhoods were associated with mental health in the children and adolescents who lived there, particularly during adolescence. Air pollution was associated with poorer mental health, especially during early childhood exposures, highlighting the need for age-appropriate interventions. While we observed weak associations between green space and mental health, research on specific types of green space and quality could offer more insights. Our findings emphasize the importance of assessing exposures at residences and schools to better capture adolescents’ broader environments. Neighborhood characteristics were associated with varying mental health outcomes from early childhood to late adolescence. Studying the associations of environmental factors in conjunction with other neighborhood characteristics is essential to avoid overestimation of their impacts.

## Notes

Supplement 1. **eFigure 1.** Flowchart of Inclusion and Exclusion Criteria **eFigure 2.** Maps of Distribution of Environmental Data in England. **eFigure 3.** The Histogram of SDQ Score **eFigure 4.** Estimated Regression Coefficients of the Main Model With PM_10_ **eFigure 5.** Estimated Regression Coefficients of the Main Model With no_2_ **eFigure 6.** Trace Plots of Model Coefficients for Fixed Effects and Time-Varying Coefficients **eTable 1.** Descriptive Statistics of Individual and Household Characteristics of the Original Population at Each Sweep **eTable 2.** Results of Sensitivity Analysis When Investigating Separately the Time-Varying Effect of Neighborhood Variables **eMethods 1.** More Details on Air Pollution Concentration Calculations **eMethods 2.** More Details on the Index of Multiple Deprivation **eMethods 3.** More Details on the Covariates Included in Our Study **eResults 1.** Model With Cumulative Exposures for Neighborhood-Level Factors **eResults 2.** Sensitivity Analyses and the Output

Supplement 2.
Data Sharing Statement
