# Supplementary material for: Neighborhood Characteristics and Mental Health From Childhood to Adolescence
Source: JAMA Netw Open. 2025 Apr 10;8(4):e254470. doi: 10.1001/jamanetworkopen.2025.4470 (PMC11986778; doi:10.1001/jamanetworkopen.2025.4470)
Supplement: Supplement 1. — eFigure 1. Flowchart of Inclusion and Exclusion Criteria eFigure 2. Maps of Distribution of Environmental Data in England. eFigure 3. The Histogram of SDQ Score eFigure 4. Estimated Regression Coefficients of the Main Model With PM10 eFigure 5. Estimated Regression Coefficients of the Main Model With no2 eFigure 6. Trace Plots of Model Coefficients for Fixed Effects and Time-Varying Coefficients eTable 1. Descriptive Statistics of Individual and Household Characteristics of the Original Population at Each Sweep eTable 2. Results of Sensitivity Analysis When Investigating Separately the Time-Varying Effect of Neighborhood Variables eMethods 1. More Details on Air Pollution Concentration Calculations eMethods 2. More Details on the Index of Multiple Deprivation eMethods 3. More Details on the Covariates Included in Our Study eResults 1. Model With Cumulative Exposures for Neighborhood-Level Factors eResults 2. Sensitivity Analyses and the Output [file jamanetwopen-e254470-s001.pdf]

Supplemental Online Content

Shoari N, Blangiardo M, Pirani M. Neighborhood characteristics and mental health from childhood to adolescence. *JAMA Netw Open*. 2025;8(4):e254470. doi:10.1001/jamanetworkopen.2025.4470

eFigure 1. Flowchart of Inclusion and Exclusion Criteria

eFigure 2. Maps of Distribution of Environmental Data in England.

eFigure 3. The Histogram of SDQ Score

eFigure 4. Estimated Regression Coefficients of the Main Model With PM10

eFigure 5. Estimated Regression Coefficients of the Main Model With NO2

eFigure 6. Trace Plots of Model Coefficients for Fixed Effects and Time-Varying Coefficients

eTable 1. Descriptive Statistics of Individual and Household Characteristics of the Original Population at Each Sweep

eTable 2. Results of Sensitivity Analysis When Investigating Separately the Time-Varying Effect of Neighborhood Variables

eMethods 1. More Details on Air Pollution Concentration Calculations

eMethods 2. More Details on the Index of Multiple Deprivation

eMethods 3. More Details on the Covariates Included in Our Study

eResults 1. Model With Cumulative Exposures for Neighborhood-Level Factors

eResults 2. Sensitivity Analyses and the Output

This supplemental material has been provided by the authors to give readers additional information about their work.

© 2025 Shoari N et al. *JAMA Network Open*.

eFigure1. The flowchart of inclusion and exclusion criteria.

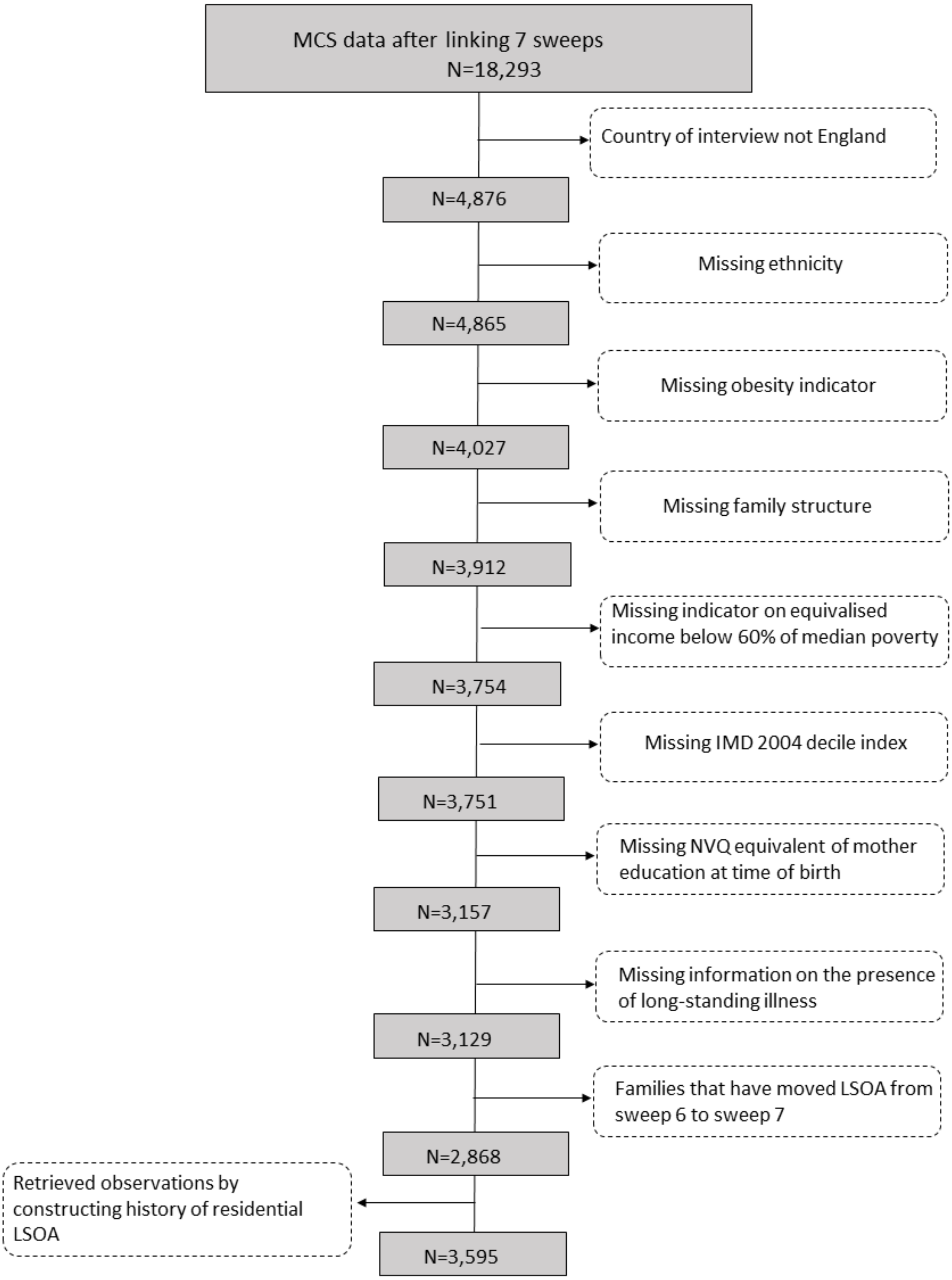

© 2025 Shoari N et al. *JAMA Network Open*.

eFigure2. Maps of distribution of environmental data a) greenness b) greenspace area with the function of public park, garden, playing field, and play spaces, c) $PM_{2.5}$, d) PM_{10}, e) NO_2 in England at the start (2000), midpoint (2008) and end (2018) of study period.

a) NDVI

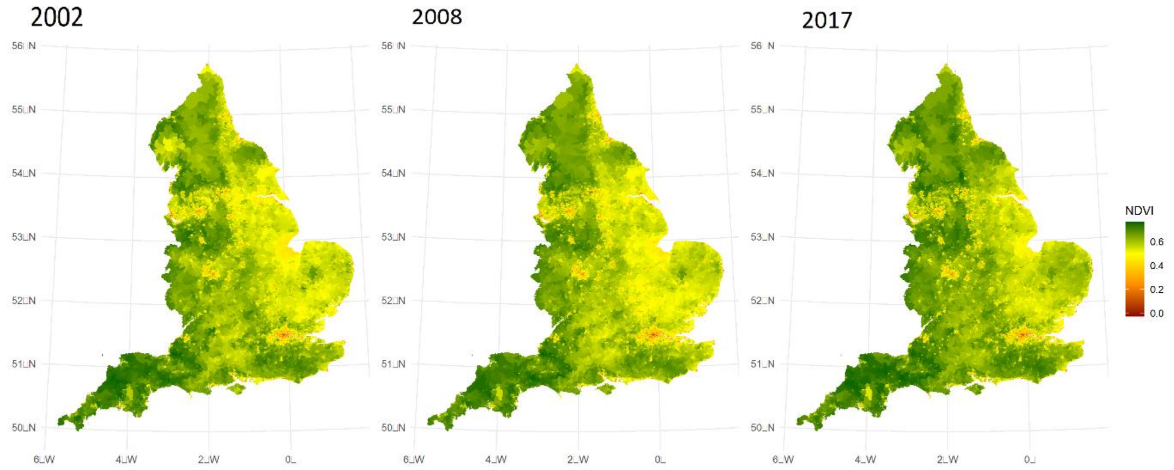

b) The area of greenspace with the function of public park, garden, playing field, and play spaces in 2017

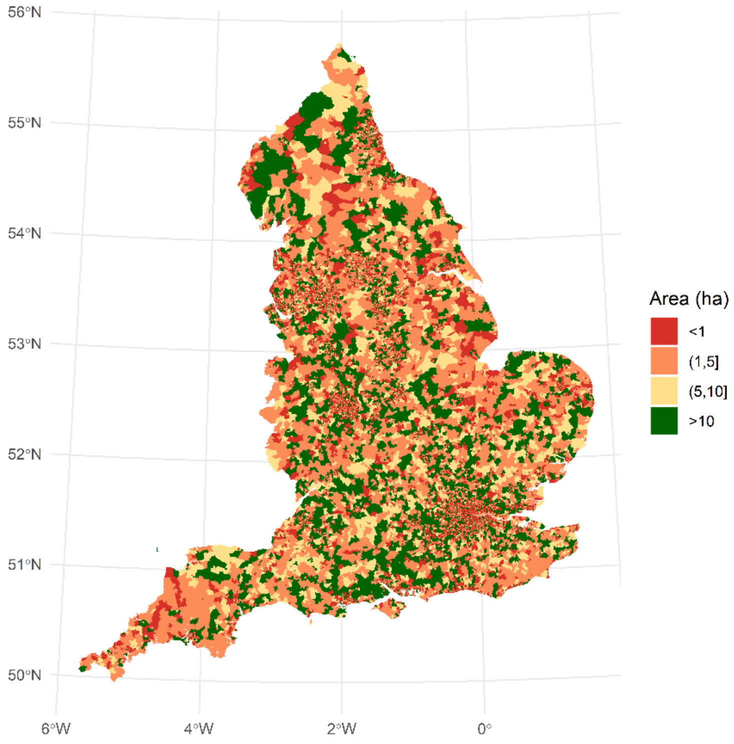

© 2025 Shoari N et al. *JAMA Network Open*.

c) $PM_{2.5}$ concentration

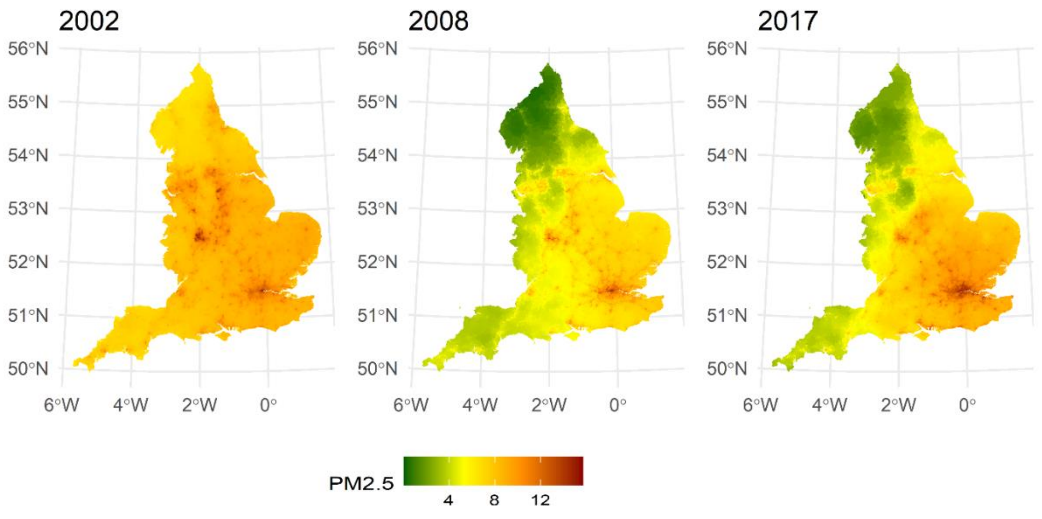

d) PM_{10} concentration

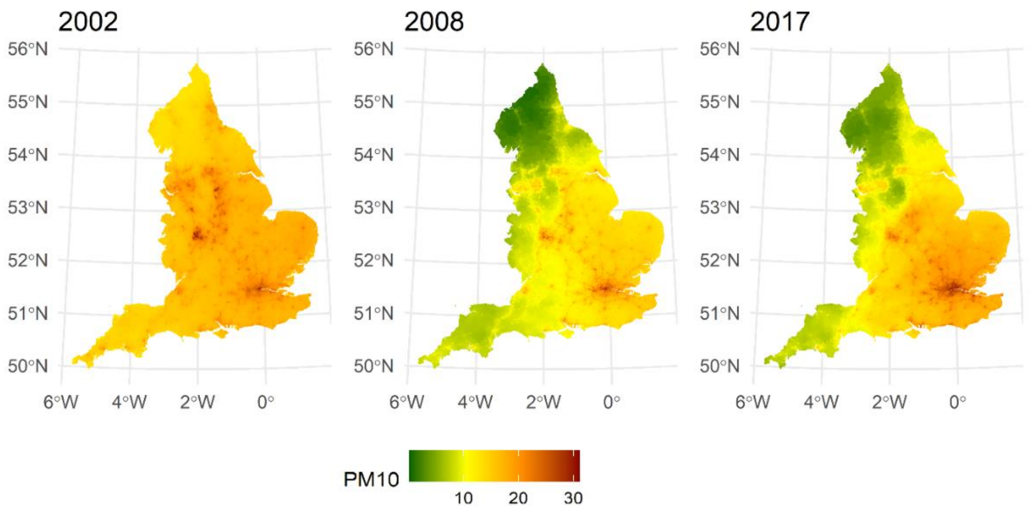

e) NO_2 concentration

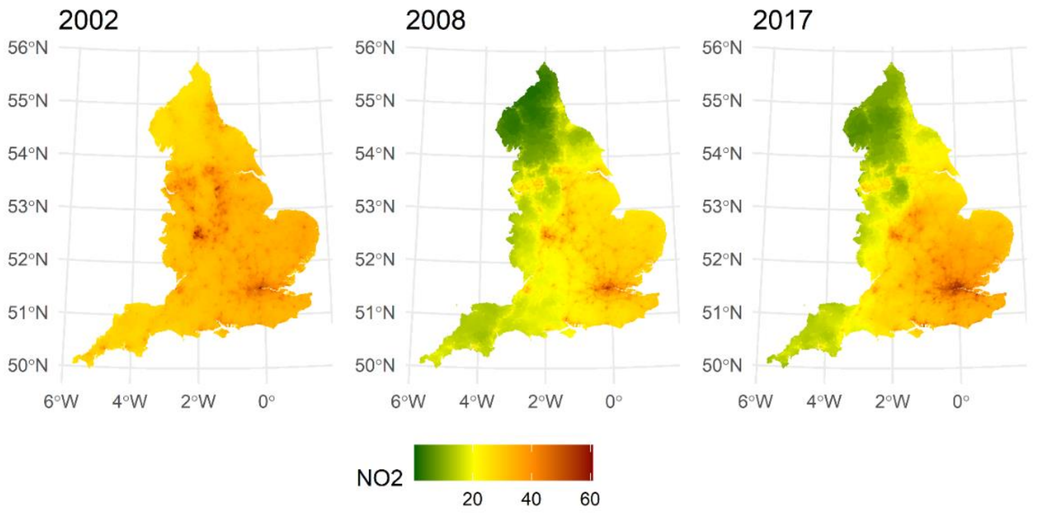

© 2025 Shoari N et al. *JAMA Network Open*.

eFigure3 The histogram of SDQ score

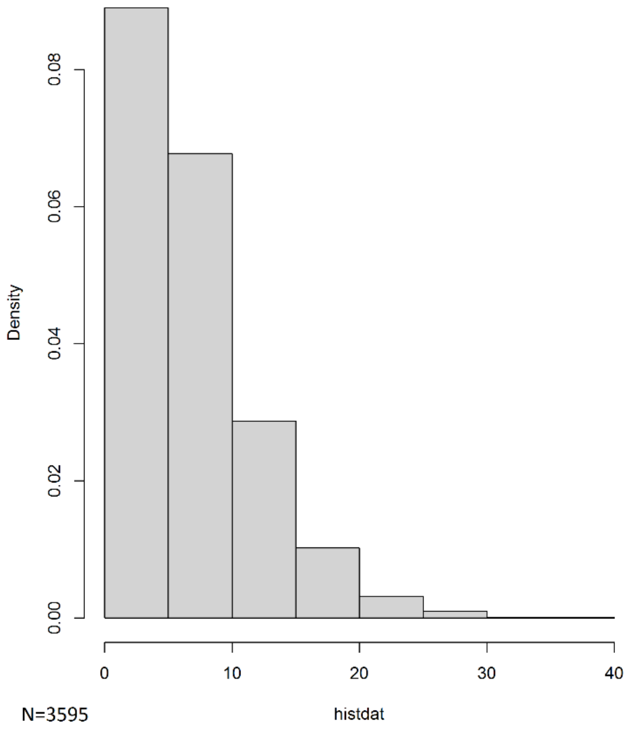

.

© 2025 Shoari N et al. *JAMA Network Open*.

eFigure 4. Estimated regression coefficients (posterior mean and 95%CrI) of the main model with PM_{10}. A): estimated coefficients for individual and household variables, B) estimated time-varying coefficients for neighbourhood variables

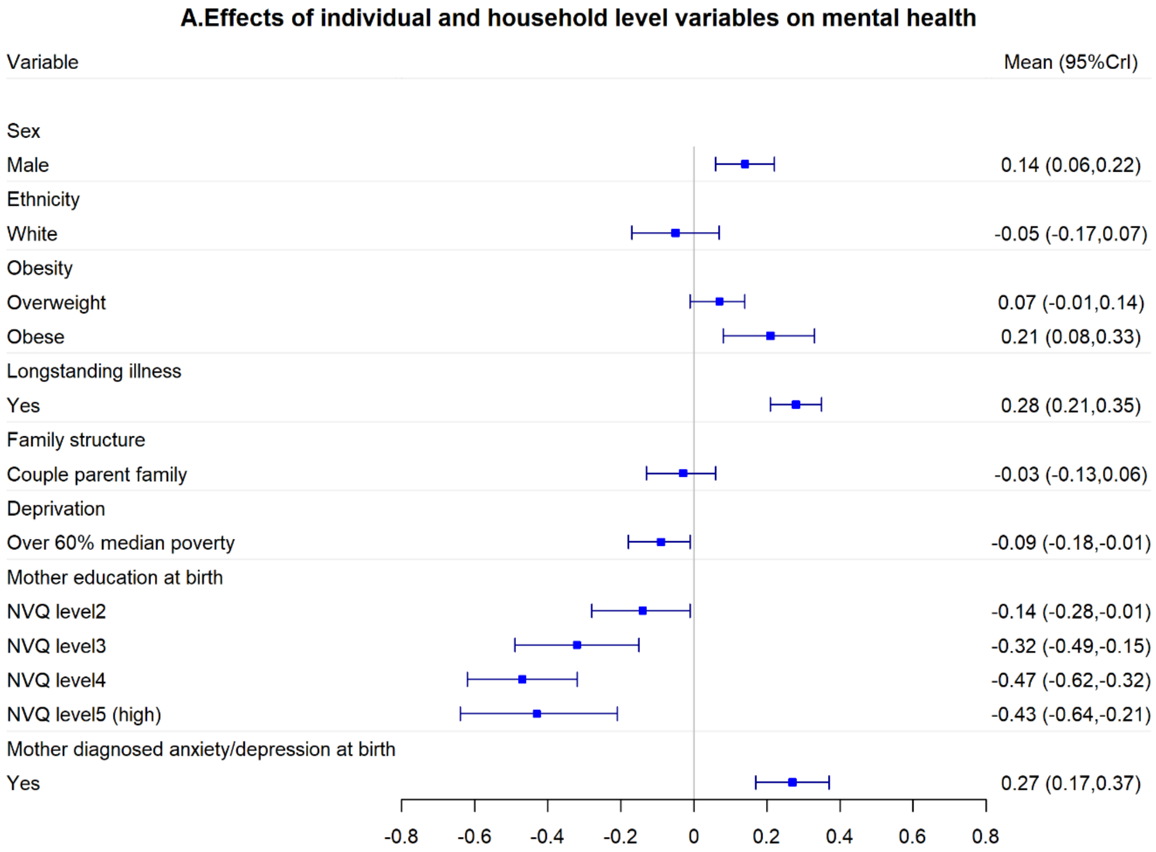

© 2025 Shoari N et al. *JAMA Network Open*.

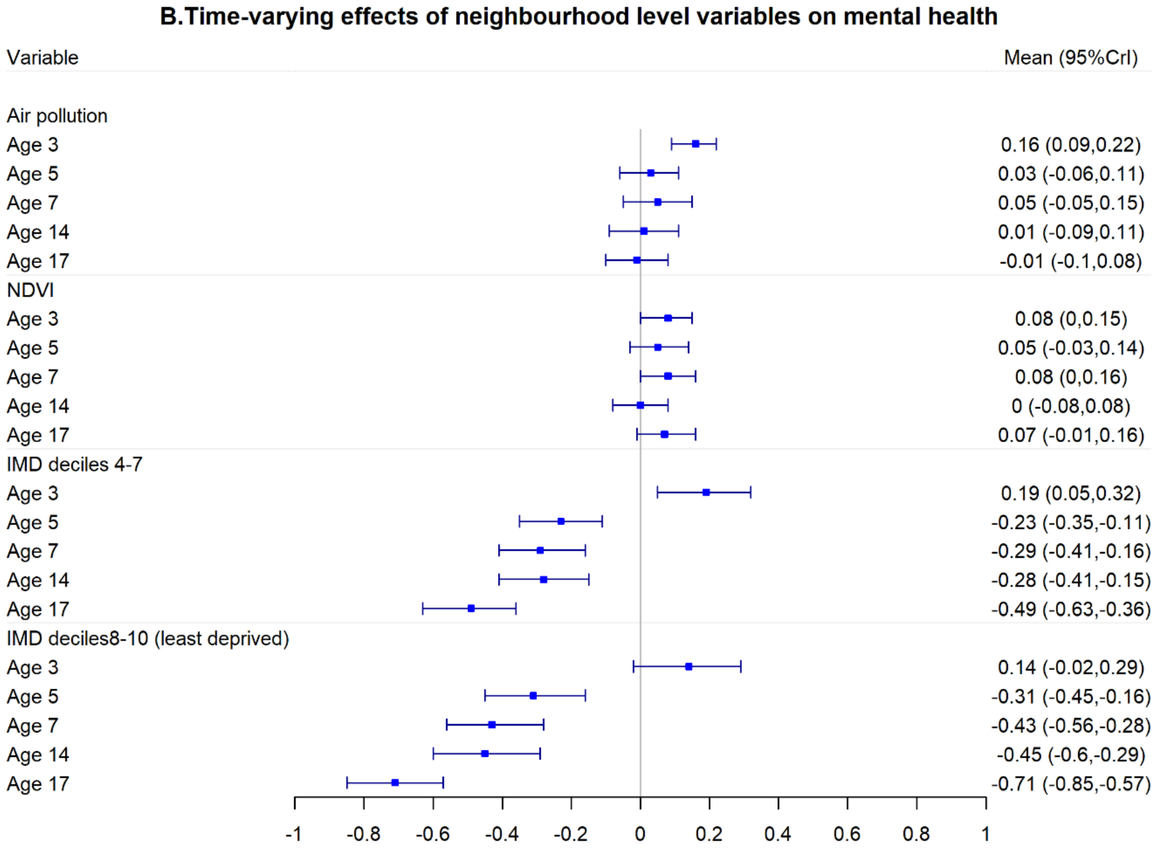

IMD: Index of Multiple Deprivation; NVQ: National Vocational Qualification; Greenness refers to NDVI.

© 2025 Shoari N et al. *JAMA Network Open*.

eFigure5. Estimated regression coefficients (posterior mean and 95%CrI) of the main model with NO_2. A): estimated coefficients for individual and household variables, B) estimated time-varying coefficients for neighbourhood variables

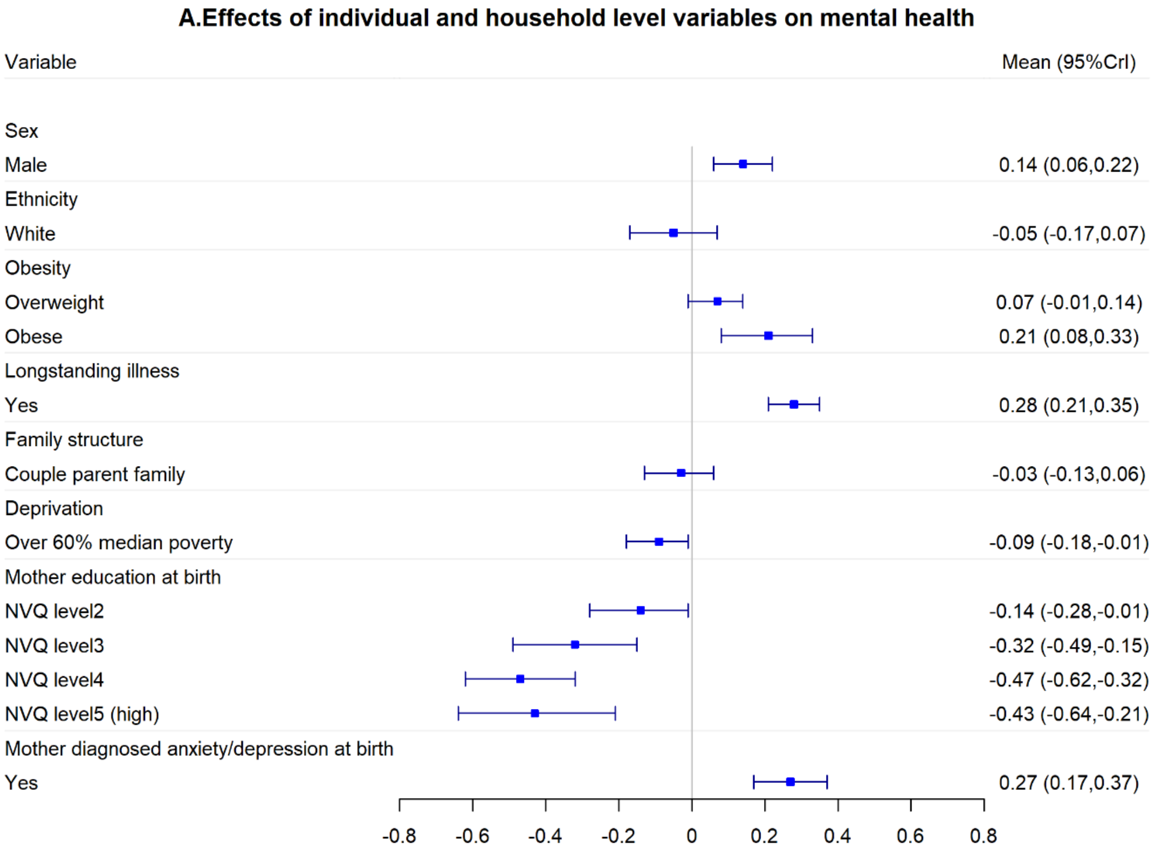

© 2025 Shoari N et al. *JAMA Network Open*.

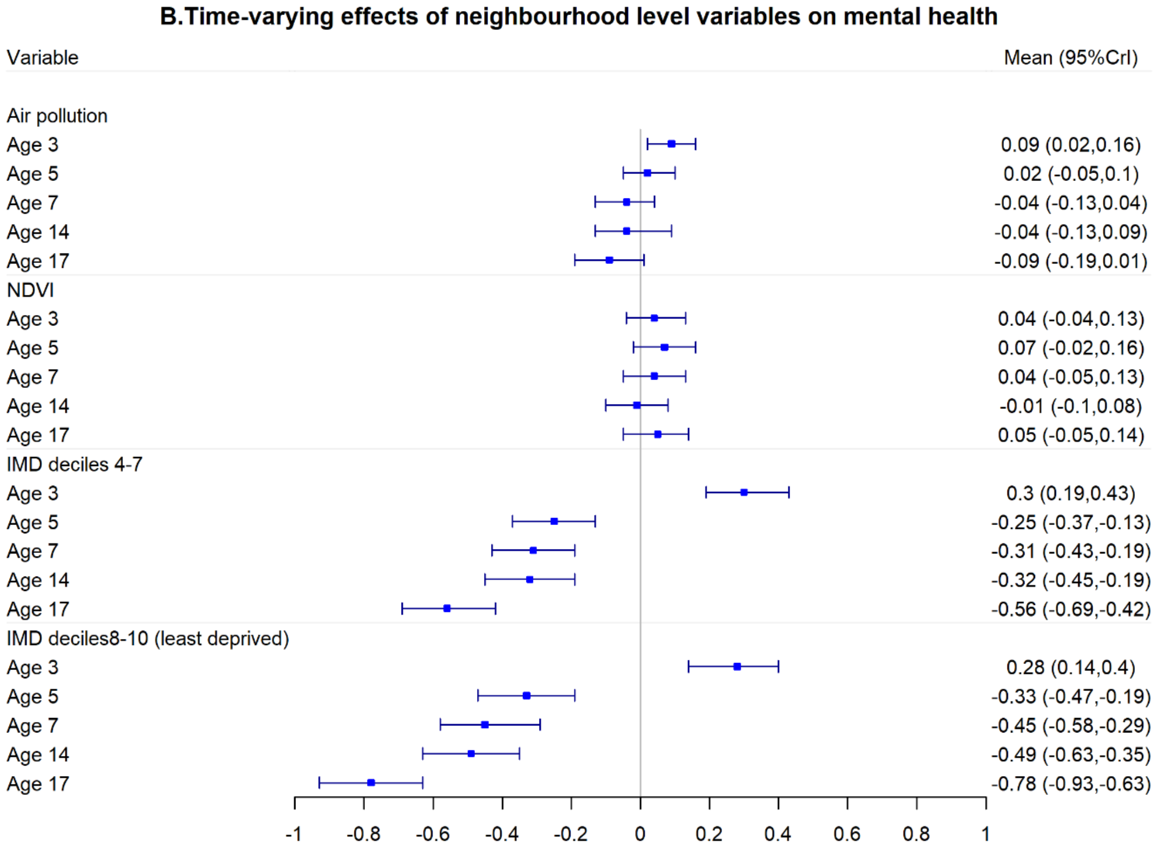

IMD: Index of Multiple Deprivation; NVQ: National Vocational Qualification. Greenness refers to NDVI

© 2025 Shoari N et al. *JAMA Network Open*.

eFigure 6 Trace plots of model coefficients for A) fixed effects (β) and time-varying coefficients (γ).

Beta.year1 refers to coefficients associated with NDVI, beta.year2 refers to coefficients associated with air pollution, and beta.year3 and beta.year4 refer to IMD deciles 4-7and IMD deciles 8-10, respectively.

A)

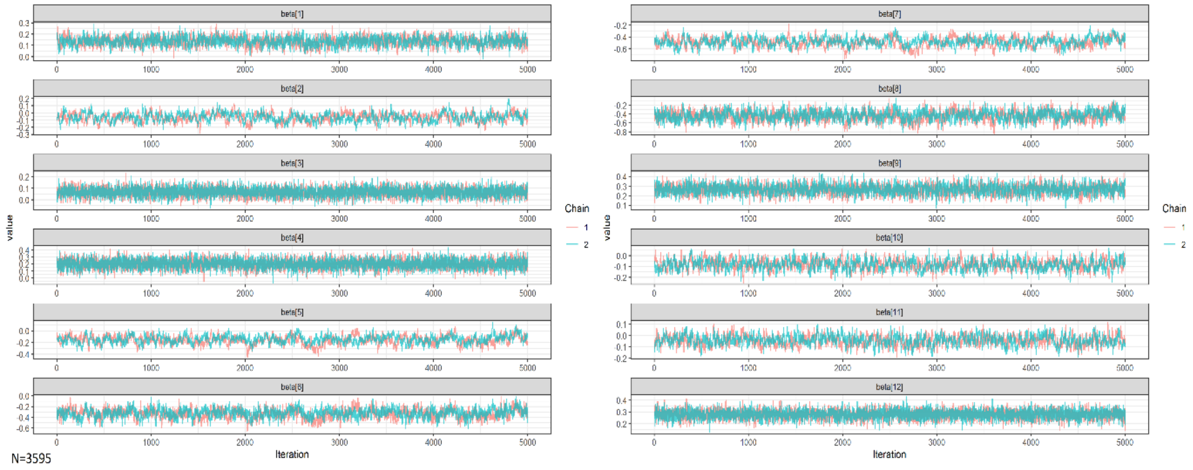

B)

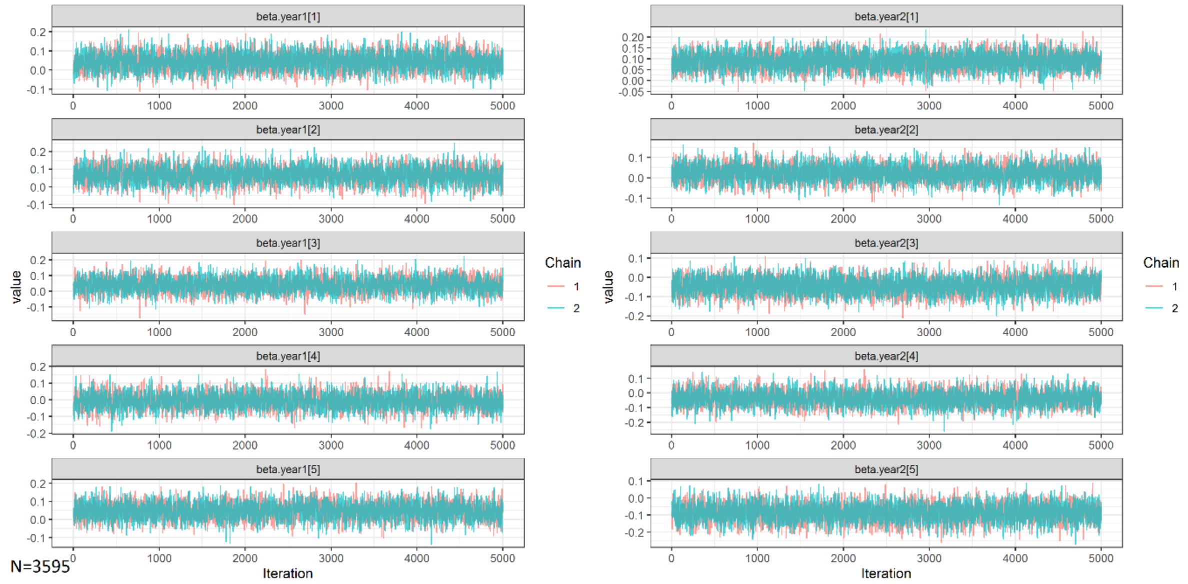

© 2025 Shoari N et al. *JAMA Network Open*.

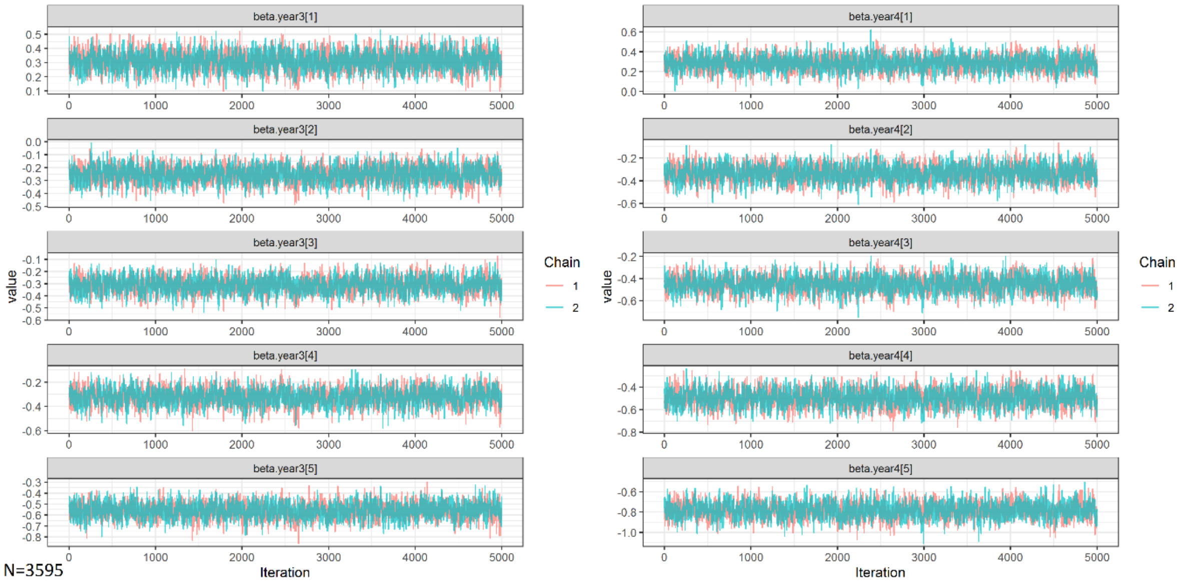

© 2025 Shoari N et al. *JAMA Network Open*.

eTable 1. Descriptive statistics of individual and household characteristics of the original population at each sweep

	MCS2 (N=10171)	MCS3 (N=9879)	MCS4 (N=8988)	MCS5 (N=8780)	MCS6 (N=7814)	MCS7 (N=7043)
**Individual characteristics**						
Total SDQ score	8.8 (6.0)	7.1 (5.3)	7.3 (5.7)	5.5 (5.5)	8.0 (6.2)	6.3 (6.2)
SDQ score components						
Emotional problems	1.2 (1.6)	1.3 (1.7)	1.5 (1.8)	1.4 (1.9)	2.0 (2.2)	1.6 (2.3)
Conduct problems	2.6 (2.2)	1.4 (1.6)	1.3 (1.6)	0.7 (1.4)	1.4 (1.7)	0.9 (1.5)
Hyperactivity/Inattention	3.6 (2.6)	3.1 (2.5)	3.2 (2.6)	2.3 (2.5)	2.9 (2.5)	2.0 (2.4)
Peer problems	1.4 (1.7)	1.1 (1.5)	1.2 (1.6)	1.2 (1.7)	1.7 (1.9)	1.4 (1.9)
Sex						
Male (%)	4692 (46.1)	4636 (46.9)	4184 (46.6)	4091 (46.6)	3628 (46.4)	3216 (45.7)
Female (%)	4537 (44.6)	4434 (44.9)	4105 (45.7)	4001 (45.6)	3595 (46.0)	3332 (47.3)
Missing (%)	942 (9.3)	809 (8.2)	699 (7.8)	688 (7.8)	591 (7.6)	495 (7.0)
Ethnicity						
White (%)	6981 (68.6)	6845 (69.3)	6304 (70.1)	6075 (69.2)	5364 (68.6)	4823 (68.5)
Non-White (%)	2223 (21.9)	2197 (22.2)	1960 (21.8)	1995 (22.7)	1837 (23.5)	1708 (24.3)
Missing (%)	967 (9.5)	837 (8.5)	724 (8.0)	710 (8.1)	613 (7.8)	512 (7.3)
Cohort member has a chronic illness						
No (%)	8465 (83.2)	7901 (80.0)	7204 (80.2)	7516 (85.6)	6424 (82.2)	5540 (78.7)
Yes (%)	1592 (15.7)	1915 (19.4)	1657 (18.4)	1188 (13.5)	1290 (16.5)	1183 (16.8)
Missing (%)	114 (1.12)	63 (<1)	127 (1.4)	76 (<1)	100 (1.3)	320 (4.5)
Obesity						
Normal (%)	7284 (71.6)	7718 (78.1)	7080 (78.8)	6190 (70.5)	5380 (68.9)	4471 (63.5)
Overweight (%)	1581 (15.5)	1451 (14.7)	1246 (13.9)	1773 (20.2)	1361 (17.4)	1200 (17.0)
Obese (%)	496 (4.9)	541 (5.5)	535 (5.95)	538 (6.1)	539 (6.9)	663 (9.4)
Missing (%)	810 (8.0)	169 (1.7)	127 (1.41)	279 (3.2)	534 (6.8)	709 (10.1)
**Household characteristics**						
Poverty						
Above 60% median poverty indicator (%)	6618 (65.1)	6355 (64.3)	6211 (69.1)	6403 (72.9)	5360 (68.6)	NA
Below 60% median poverty indicator (%)	3412 (33.5)	3442 (34.8)	2763 (30.7)	2377 (271.)	2447 (31.3)	NA
Missing (%)	141 (1.4)	82 (<1)	14 (<1)	0 (0)	10 (<1)	NA
OECD[1] equivalised household income (£)	323.3 (229.3)	342.1 (225.4)	379.5 (237.3)	406.2 (185.4)	408.8 (186.6)	NA
Family status						
Two parent/carer (%)	8324 (81.8)	7925 (80.2)	7111 (79.1)	6654 (75.8)	5839 (74.7)	4696 (66.7)
One parent/carer (%)	1780 (17.5)	1953 (19.8)	1877 (20.9)	2126 (24.2)	1974 (25.3)	1909 (27.1)
Missing (%)	67 (<1)	10 (<1)	0 (0)	0 (0)	10 (<1)	438 (6.2)
Mother ever diagnosed with mental health issues at birth						
No (%)	7138 (70.2)	6996 (70.8)	6391 (71.1)	6228 (70.9)	5597 (71.6)	5104 (72.5)

© 2025 Shoari N et al. *JAMA Network Open*.

Yes (%)	2073 (20.4)	2048 (20.7)	1878 (20.9)	1846 (21.0)	1610 (20.6)	1431 (20.3)
Missing (%)	960 (9.4)	835 (8.4)	719 (8.0)	706 (8.0)	607 (7.8)	508 (7.2)
Mother education at birth- National Vocational Qualification (NVQ)						
NVQ level 1 (%)	1068 (10.5)	1023 (10.4)	914 (10.2)	906 (10.3)	788 (10.1)	660 (9.4)
NVQ level 2 (%)	3046 (29.9)	2993 (30.3)	2766 (30.8)	2651 (30.2)	2300 (29.4)	2069 (29.4)
NVQ level 3 (%)	784 (7.7)	767 (7.8)	723 (8.0)	706 (8.0)	626 (8.0)	579 (8.2)
NVQ level 4 (%)	2025 (19.9)	2005 (20.3)	1882 (20.9)	1845 (21.0)	1710 (21.9)	1602 (22.7)
NVQ level 5 (%)	320 (3.1)	320 (3.2)	296 (3.3)	283 (3.2)	278 (3.6)	279 (4.0)
Missing (%)	2928 (28.8)	2771 (28.0)	2407 (26.8)	1389 (27.2)	2112 (27.0)	1854 (26.3)
Note: Total SDQ score and OECD equivalised household income are presented as mean (standard deviation) and categorical variables are presented as number (%).						
[1]OECD stands for "Organization for Economic Co-operation and Development".						

© 2025 Shoari N et al. *JAMA Network Open*.

eTable2 shows the results of model when neighbourhood-level factors were studied in isolation. Each column presents coefficients from separate models, each adjusted for individual and household covariates. Models differ by the environmental covariate included: PM2.5, NDVI, area of greenspace, or IMD.

eTable 2. Results of sensitivity analysis when investigating separately the time-varying effect of neighbourhood variables (N=3595)

Variable	Mean [95% CrI]	Mean [95% CrI]	Mean [95% CrI]	Mean [95% CrI]
	NDVI only model	$PM_{2.5}$ only model	IMD only model	Greenspace area only model
Individual and household level characteristics				
Sex (Male)	**0.13 [0.06,0.21]**	**0.13 [0.05,0.21]**	**0.14 [0.06,0.21]**	**0.13 [0.05,0.21]**
Ethnicity (White)	-0.05 [-0.16,0.07]	0.01 [-0.13,0.10]	-0.05 [-0.17,0.06]	**-0.12 [-0.23,-0.01]**
Obesity				
Overweight	0.07 [-0.01,0.14]	**0.08 [0.01,0.16]**	0.06 [-0.01,0.14]	0.07 [-0.01,0.14]
Obese	**0.17 [0.05,0.30]**	**0.23 [0.11,0.35]**	**0.21 [0.08,0.33]**	**0.18 [0.05,0.30]**
Mother education				
NVQ level 2	**-0.18 [-0.33,-0.04]**	**-0.20[-0.34,-0.06]**	-0.14 [-0.28, 0.00]	**-0.19 [-0.34,-0.05]**
NVQ level 3	**-0.37 [-0.54,-0.20]**	**-0.40 [-0.57,-0.23]**	**-0.32 [-0.48,-0.15]**	**-0.39 [-0.56,-0.22]**
NVQ level 4	**-0.54 [-0.68,-0.40]**	**-0.57 [-0.71,-0.43]**	**-0.47 [-0.61,-0.32]**	**-0.57 [-0.71,-0.43]**
NVQ level 5	**-0.50 [-0.71,-0.29]**	**-0.54 [-0.75,-0.33]**	**-0.43 [-0.64,-0.22]**	**-0.52 [-0.73,-0.31]**
Mother been diagnosed with anxiety or depression after birth (Yes)	**0.27 [0.17,0.37]**	**0.28 [0.18,0.38]**	**0.27 [0.17,0.36]**	**0.27 [0.17,0.38]**
Over 60% median poverty threshold	**-0.18 [-0.28,-0.09]**	**-0.15 [-0.24,-0.06]**	-0.09 [-0.18,0.00]	**-0.20 [-0.29,-0.11]**
Couple parent family	0.08 [-0.01,0.17]	0.02[-0.11,0.07]	-0.03 [-0.12,0.06]	0.08 [-0.02,0.17]
Cohort member with longstanding illness (Yes)	**0.27 [0.20,0.35]**	**0.28 [0.21,0.35]**	**0.28 [0.21,0.35]**	**0.27 [0.20,0.35]**
Neighbourhood-level characteristics				
NDVI (age 3)	-0.06 [-0.12,0.00]	-	-	-
NDVI (age 5)	**-0.06 [-0.12,-0.01]**	-	-	-
NDVI (Age 7)	**-0.07 [-0.13,-0.01]**	-	-	-
NDVI (age 14)	**-0.15 [-0.21,-0.09]**	-	-	-
NDVI (age 17)	**-0.11 [-0.18,-0.05]**	-	-	-
Area greenspace (age 3)	-	-	-	0.01 [-0.05,0.05]
Area greenspace (age 5)	-	-	-	0.01 [-0.05,0.05]
Area greenspace (Age 7)	-	-	-	-0.01 [-0.08,0.06]
Area greenspace (age 14)	-	-	-	-0.04 [-0.10,0.03]
Area greenspace (age 17)	-	-	-	-0.02 [-0.09,0.05]
$PM_{2.5}$ (age 3)	-	**0.35 [0.30,0.39]**	-	-
$PM_{2.5}$ (age 5)	-	**0.11 [0.04,0.17]**	-	-
$PM_{2.5}$ (Age 7)	-	**0.12 [0.05,0.20]**	-	-
$PM_{2.5}$ (age 14)	-	**0.13 [0.06,0.19]**	-	-
$PM_{2.5}$ (age 17)	-	**0.21 [0.15,0.27]**	-	-
IMD 4-7 (age 3)	-	-	**0.32 [0.20,0.44]**	-
IMD 4-7 (age 5)	-	-	**-0.27 [-0.38,-0.15]**	-
IMD 4-7 (age 7)	-	-	**-0.34 [-0.46, -0.22]**	-
IMD 4-7 (age 14)	-	-	**-0.34 [-0.46,-0.22]**	-
IMD 4-7 (age 17)	-	-	**-0.53[-0.65,-0.41]**	-
IMD 8-10 (age 3)	-	-	**0.27 [0.14,0.40]**	-
IMD 8-10 (age 5)	-	-	**-0.35 [-0.48,-0.22]**	-

© 2025 Shoari N et al. *JAMA Network Open*.

IMD 8-10 (age 7)	-	-	**-0.47 [-0.60,-0.34]**	-
IMD 8-10 (age 14)	-	-	**-0.53 [-0.65,-0.40]**	-
IMD 8-10 (age 17)	-	-	**-0.75 [-0.87,-0.62]**	-

NVQ: National Vocational Qualification; NDVI: Normalized Difference Vegetation Index; $PM_{2.5}$: Particulate matter with diameter ≤2.5 µm
IMD: Index of Multiple Deprivation

© 2025 Shoari N et al. *JAMA Network Open*.

eMethod 1. More details on air pollution concentration calculations

Annual averages of air pollution concentrations were estimated through a hybrid approach using data from monitoring stations and dispersion models, at a spatial resolution of 1 km x 1 km. We calculated the annual average air pollution exposures as the average of concentration points within each LSOA. PM_{10} concentrations (2001-2003) used Tapered Element Oscillating Microbalance (TEOM) analysers, adjusted by a factor of 1.3 for comparability with subsequent years (Department of the Environment).

Department of the Environment, Transport and the Regions. Assistance with the Review and Assessment of PM_{10} Concentration in Relation to the Proposed EU Stage 1 Limit Value. 1999.

eMethod2. More details on the Index of Multiple Deprivation (IMD)

The Index of Multiple Deprivation (IMD) is a comprehensive measure that assesses area-level socioeconomic status. It is composed of multiple weighted domains, including education, health, income, employment, crime, barriers to housing and services, and living environment.

The Income Deprivation Domain measures the proportion of individuals in an area experiencing income deprivation, including those out of work, those with low earnings meeting means-tested criteria, and specific groups such as families receiving benefits, tax credits, or asylum support.

The Employment Deprivation Domain measures the proportion of working-age individuals involuntarily excluded from the labor market due to unemployment, illness, disability, or caring responsibilities, as reflected by claims for jobseeker, incapacity, carer, and related benefits.

The Education, Skills and Training Deprivation Domain measures the lack of attainment and skills in the local population.

The Health Domain measures morbidity, disability, and premature mortality to provide an overall assessment of health status in the population.

The Crime Domain assesses the likelihood of personal and property victimization within a local area.

The Barriers to Housing and Services Domain evaluates physical and financial accessibility to housing and services, encompassing geographical proximity and housing-related challenges like affordability and homelessness.

The Living Environment Deprivation Domain evaluates local environmental quality through two sub-domains: housing quality ('indoors') and factors like air quality and road traffic accidents ('outdoors')

Deciles of IMD were not determined based on the distribution within our study population; they were derived from national IMD scores to ensure comparability across the broader population.

eMethod 3. More details on the covariates included in our study

We included several individual-, household-, and neighbourhood-level covariates based on existing literature and data availability. Data for these covariates were primarily collected through parent-reported questionnaires and administrative linkages as part of the Millennium Cohort Study (MCS).

At the individual level, sex and ethnicity (categorized as White versus non-White) were reported by parents during the initial survey sweeps. Obesity status was derived from body mass index (BMI) calculated using height and weight measurements taken during home visits. Longstanding illness was based on parent-reported information about whether the cohort member had any chronic health condition or disability that had lasted or was expected to last for at least a year.

Household-level variables were also gathered through parent questionnaires. Poverty status was determined using the Organisation for Economic Co-operation and Development (OECD) equivalised income threshold of 60% of the median household income. Maternal education was categorized according to National Vocational Qualification (NVQ) levels, as reported by the primary caregiver at birth. Maternal mental health was assessed based on parental self-report of ever being diagnosed with depression or serious anxiety at the time of the child's birth. Family structure (single-parent or two-parent households) was recorded based on household composition at each sweep.

At the neighbourhood level, rural or urban classification was based on the Office for National Statistics Rural-Urban Classification, linked to the postcode or Lower Layer Super Output Area (LSOA) of the cohort member's residence at each sweep.

eResults1. Model with cumulative exposures for neighbourhood-level factors

We modelled the log-transformed SDQ to examine the associations of cumulative exposure to neighbourhood-level factors and compared these with the estimated coefficients from the model with age-specific terms. Cumulative exposure to air pollution and NDVI was calculated as the average up to each age. For IMD, which is categorized into three groups—most deprived (deciles 1-3), moderately deprived (deciles 4-7), and least deprived (deciles 8-10)—we calculated the number of times an individual resided in each category. The estimated coefficients are reported below for PM2.5; other air pollutants are not reported as their results were very similar.

	Mean [95%CrI]
Male (N=1826)	0.14 [0.06,0.21]
White (N=3012)	0.01 [-0.12,0.13]
Obesity	
Overweigh (N=1485)t	0.09 [0.01,0.16]
Obese (N=533)	0.24 [0.11,0.37]
Mother education	
NVQ level 2	-0.14 [-0.28,0.01]
NVQ level 3	-0.31 [-0.48,-0.13]
NVQ level 4	-0.46 [-0.59,-0.30]
NVQ level 5	-0.41 [-0.62,-0.20]
Mental health	0.25 [0.16,0.36]
Poverty	-0.05 [-0.14,0.03]
Parent status	-0.08 [-0.17,0.01]
Illness	0.26 [0.19,0.33]
Cumulative NDVI	0.00 [-0.05,0.06]
Cumulative PM2.5	0.04 [-0.01,0.09]
Cumulative number of times in IMD 1-3	-0.08 [-0.11,-0.04]
Cumulative number of times in IMD 4-7	-0.16 [-0.19,-0.14]
Cumulative number of times in IMD 8-10	-0.22 [-0.25,-0.19]

eResults2. Sensitivity analyses and the output

All models exhibited no collinearity (all VIF <3.0), except when including interaction between air pollution and household income, air pollution and ethnicity, neighbourhood greenness and family status, greenness and ethnicity. Sensitivity analyses generally supported the main analysis results, with specifications and outputs summarized below:

Sensitivity analysis specification	Output
Including only individuals with complete SDQ score and covariates for all sweeps	Estimates consistent with the original model
Including only individuals with complete residential histories	Estimates consistent with the original model
Assuming a Normal distribution prior for the sigma parameter (τ^{-2})	Estimates consistent with the original model
Restricting the analysis to urban areas	Estimates consistent with the original model
Including teacher-reported SDQscore in sweep 5	Minor changes noted in individual and household covariates; however, neighbourhood-level estimates, particularly at age 11 (Sweep 5), diverged from trends observed in other age groups. For consistency, Sweep 5 was excluded in final analyses.
Investigating the time-varying effect of neighbourhood-level covariate separately while keeping other variables the same	Results are reported in eTable1

© 2025 Shoari N et al. *JAMA Network Open*.

Investigate the independence of time-varying coefficients across time points and exposures (assuming a prior Normal(0,0.001) for $\gamma_{t,k}$	Estimates consistent with the original model; the WAIC increased to 64,767.
Treating SDQ score as counts and model them with Poisson distribution	Estimates consistent with the original model; the WAIC increased to 90,966.

© 2025 Shoari N et al. *JAMA Network Open*.
