# Supplementary material for: Neighborhood Characteristics and Mental Health From Childhood to Adolescence
Source: JAMA Netw Open. 2025 Apr 10;8(4):e254470. doi: 10.1001/jamanetworkopen.2025.4470 (PMC11986778; doi:10.1001/jamanetworkopen.2025.4470)
Supplement: Supplement 2. — Data Sharing Statement [file jamanetwopen-e254470-s002.pdf]

Data Sharing Statement

Shoari. Neighborhood Characteristics and Mental Health From Childhood to Adolescence. *JAMA Netw Open*. Published April 10, 2025. doi:10.1001/jamanetworkopen.2025.4470

Data
Data available: No

Additional Information
Explanation for why data not available: Individual-level data can be requested through UK Data Service https://beta.ukdataservice.ac.uk/datacatalogue/series/series?id=2000031
